# Molecular Structure, Expression and Role of TAFA4 and its Receptor FPR1 in the Spinal Cord

**DOI:** 10.3389/fcell.2022.911414

**Published:** 2022-05-31

**Authors:** Sipin Zhu, Xiaoyong Hu, Samuel Bennett, Yuliang Mai, Jiake Xu

**Affiliations:** ^1^ Department of Orthopaedics, The Second Affiliated Hospital and Yuying Children’s Hospital of Wenzhou Medical University, Wenzhou, China; ^2^ Molecular Lab, School of Biomedical Sciences, University of Western Australia, Perth, WA, Australia; ^3^ Guangdong Provincial Key Laboratory of Industrial Surfactant, Guangdong Research Institute of Petrochemical and Fine Chemical Engineering, Guangdong Academy of Sciences, Guangzhou, China

**Keywords:** TAFA4, FAM19A, FPR1, spinal cord, dorsal root ganglia, chemotaxis, macrophages, signaling

## Abstract

TAFA chemokine like family member 4 (TAFA4, also named FAM19A4) is a member of the TAFA chemokine like ligand or FAM19A family, which includes TAFA1, TAFA2, TAFA3, TAFA4, and TAFA5 (or FAM19A1, FAM19A2, FAM19A3, FAM19A4, and FAM19A5). They are also referred to as neurokines and are involved in the regulation of a diverse range of cellular processes, including chemotaxis of macrophages, phagocytosis, and release of reactive oxygen species (ROS). TAFA4 is a marker of C-low-threshold mechanoreceptors and is expressed predominantly in nociceptors, such as dorsal root ganglia (DRG). TAFA4 has been implicated in the sensory perception of pain in the spinal cord. Mice with deficiency of TAFA4 demonstrate altered excitability in lamina IIi neurons in DRG in addition to increased mechanical and chemical nociception following inflammation or injury. As a secreted protein, TAFA4 binds to cell surface receptor formyl peptide receptor 1 (FPR1), a G protein-coupled receptor to mediate the chemoattraction of macrophages, phagocytosis, and the inflammatory profile of macrophages. It also interacts with cell surface neurexin to mediate signalling across the synapse. Further understanding the mechanisms by which this conserved protein family regulates diverse biological processes such as in neuronal functions, inflammation, and tissue fibrosis will help to design therapeutic targets for the treatment of TAFA related diseases such as spinal cord injury and neuro-inflammatory disorders.

## Introduction

Chemokine like ligands have pleiotropic functions in diverse tissues such as peripheral nerves and brain. TAFA or FAM19A proteins represent a new class of chemokine like ligands that are revealed as neuron-derived secretory proteins, or neurokines, and have been implicated in the regulation of immune responses within the central nervous system (CNS). The TAFA family comprises five members of ligands, namely TAFA1, TAFA2, TAFA3, TAFA4, and TAFA5 (also known as FAM19A1, FAM19A2, FAM19A3, FAM19A4, and FAM19A5, respectively). The TAFA family genes encode approximately 12–15 kDa secretory proteins, which are expressed in the central and peripheral nervous tissues. TAFAs were found to act as neutrophil chemotactic factors (Postma et al., 2004; Prat et al., 2009), and to coordinate with other chemokines to recruit immune cells and regulate their activity in the CNS (Tom Tang et al., 2004; Sarver et al., 2021). TAFAs also act as neurokines to regulate nervous cell and tissue repair and regeneration after brain injury (Tom Tang et al., 2004; Sarver et al., 2021). Among TAFA ligands, TAFA4, also called FAM19A4 is a secreted chemokine-like protein of 12 kDa weight, which has been relatively well studied, with research implicating its importance for the detection of cervical cancer (Delfini et al., 2013; Wang et al., 2015; Luttmer et al., 2016b).

The critical role of TAFA4 in cancer diagnosis continues to be a vital area of research. For instance, analysis by Quantitative Methylation-Specific PCR shows that methylation of the FAM19A4 (or TAFA4) gene is a putative marker for patients with cervical intra-epithelial neoplasia (Steenbergen et al., 2013; Luttmer et al., 2016a; Luttmer et al., 2016b; De Strooper et al., 2016; Lorincz, 2016; De Strooper et al., 2018; Vink et al., 2021). FAM19A4 gene DNA methylation therefore appears to be an attractive marker for the detection of advanced cervical carcinoma lesions in hrHPV-positive patients (De Strooper et al., 2014; Vink et al., 2020; Bonde et al., 2021). DNA methylation of FAM19A4 could also be a potential biomarker of lung cancer (Hubers et al., 2017; Liu et al., 2017). Further studies are required to confirm the involvement of DNA methylation of TAFA4/FAM19A4 in the development of cancers in the cervix and lung, and to add DNA methylation of TAFA4/FAM19A4 as an additional diagnostic marker to improve specificity and sensitivity.

This review discusses the molecular structures, expression, and emerging role of TAFA4 receptor signalling with a focus on the spinal cord system and neuronal disorders. We survey comparative structure analyses of TAFA4 with other TAFA family members, and their putative receptors, formyl peptide receptor 1 (FPR1, also called N-formylpeptide chemoattractant receptor, N-formyl peptide receptor, fMet-Leu-Phe receptor; fMLF-R; fMLP receptor; FMLP) and FPR2. Understanding the pathophysiology of TAFAs in tissue tropism will facilitate the design of novel therapeutic targets for related diseases.

## Molecular Structure and Expression of TAFA4

**FIGURE 1:**
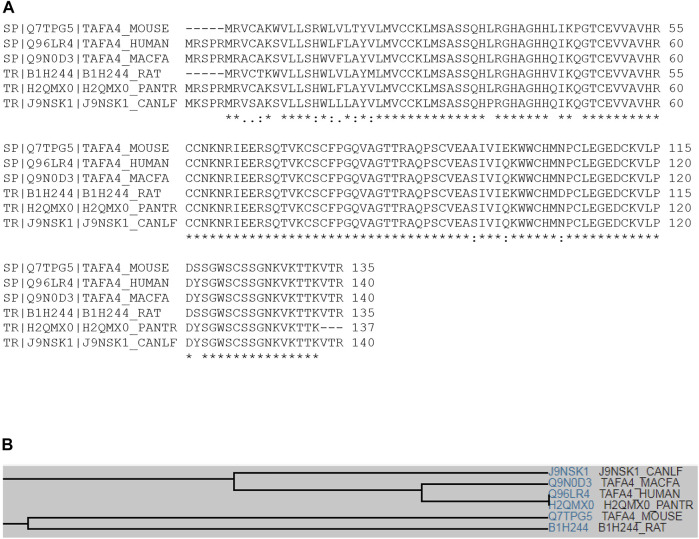
Multiple sequence alignment of TAFA4 in various species. **(A)** Multiple sequence alignment analyses show that TAFA4 shares sequence identity and similarity with TAFA4 homologues among various species including human, mouse, cynomolgus monkey, rat, chimpanzee, and dog (https://www.uniprot.org/align). **(B)** A family tree of TAFA4 homologue proteins is elucidated.

**FIGURE 2:**
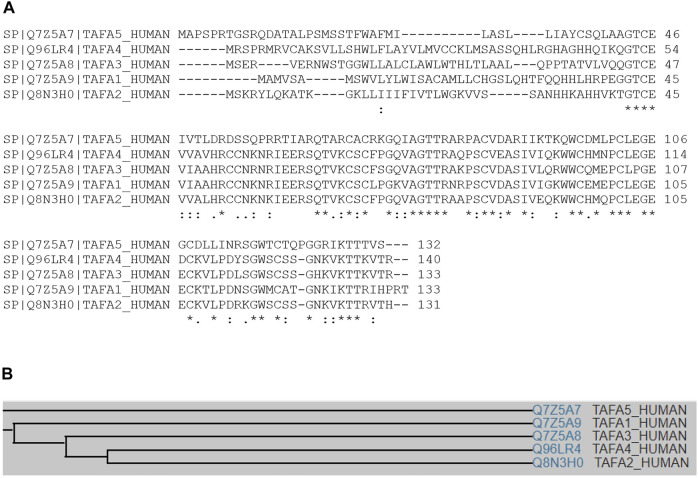
Multiple sequence alignment of TAFA4 among family members. **(A)** Multiple sequence analysis demonstrates that human TAFA1, TAFA2, TAFA3, TAFA4, and TAFA5 shares significant degree of amino acid sequence identity, especially at their C terminal regions. **(B)** Family tree of human TAFA1, TAFA2, TAFA3, TAFA4, and TAFA5.

Multiple sequence alignment analyses show that TAFA4 (or FAM19A4) displays conserved sequence identity among species, including mouse, human, cynomolgus monkey, rat, chimpanzee, and dog (Figure 1A), with a common family tree (Figure 1B) (UniPort Consortium, 2021). TAFA4 shows sequence homology with TAFA1, 2, 3 and 5 by multiple sequence alignment analyses, which demonstrate that TAFAs have a conserved CX7CCX13CXCX14CX11CX4CX5CX10C motif, except TAFA5, which has a CX7DSX13CXCX14CX11CX4CX5CX10C motif (Figure 2A), and TAFAs share a common family tree (Figure 2B) (UniPort Consortium, 2021).

**FIGURE 3:**
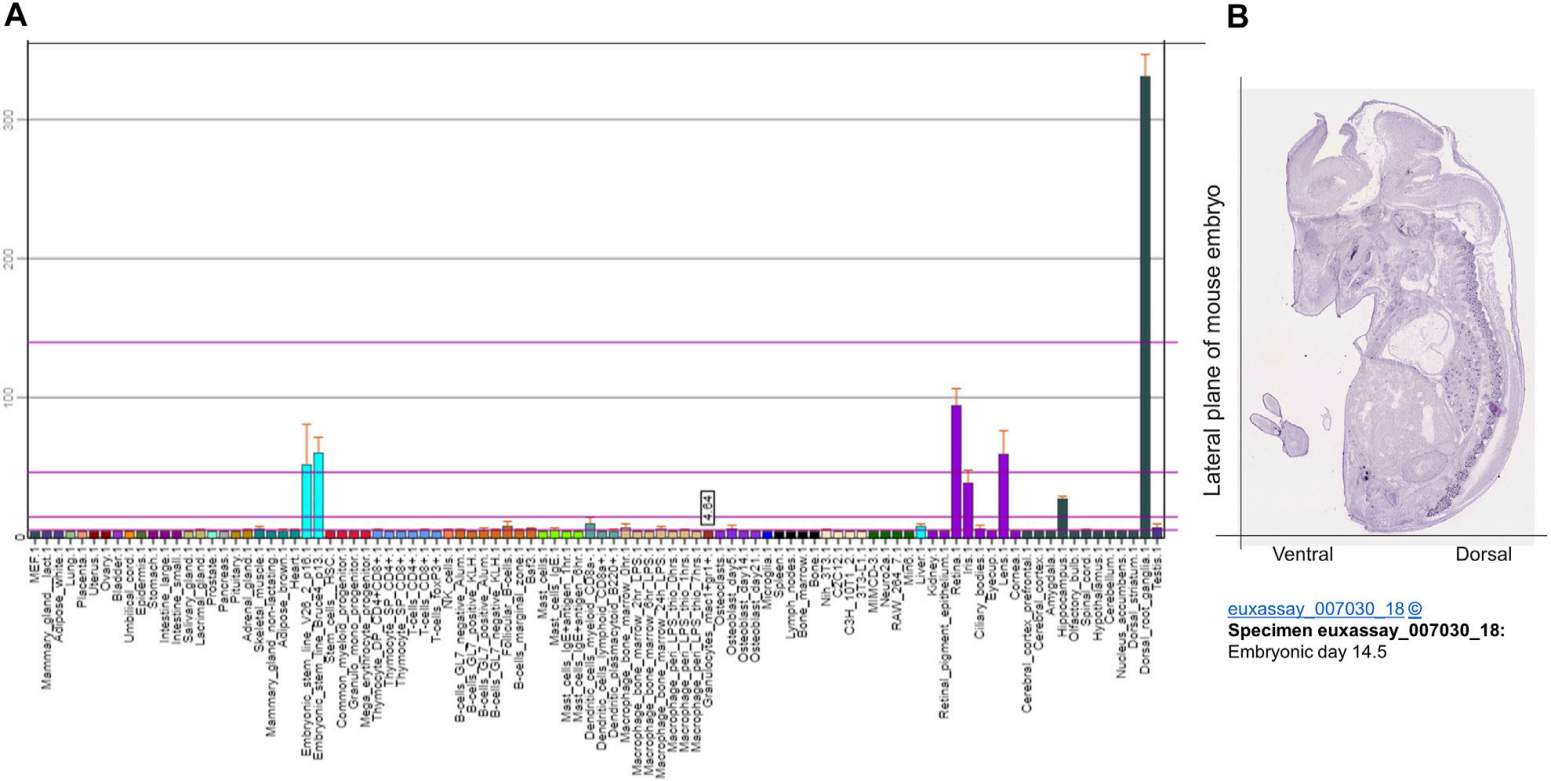
Bioinformatic analysis of TATF 4 gene expression. **(A)** Gene expression of TAFA4 by BioGPS analyses (http://biogps.org/) and **(B)** by Euxassay analysis of embryonic tissue of day 14.5 (http://www.informatics.jax.org/image/MGI:4517366).

Gene expression profile analyses by BioGPS revealed that TAFA4 mRNA is most abundantly expressed in dorsal root ganglia (DRG), followed by retina, lens, embryonic stem cells, and iris and hypothalamus in mouse (Figure 3A) (Wu et al., 2009). Consistently, gene expression analyses by Euxassay showed that TAFA4 mRNA is most abundantly expressed in DRG of mouse embryonic tissue of day 14.5 (Figure 3B). In line with this, TAFA4 was reported to be expressed in the thalamus of the central nervous system (Tom Tang et al., 2004). TAFA4 expression is also induced by lipopolysaccharide (LPS) in monocytes and macrophages, particularly in polarized M1 macrophages (Wang et al., 2015; Li et al., 2018).

**FIGURE 4:**
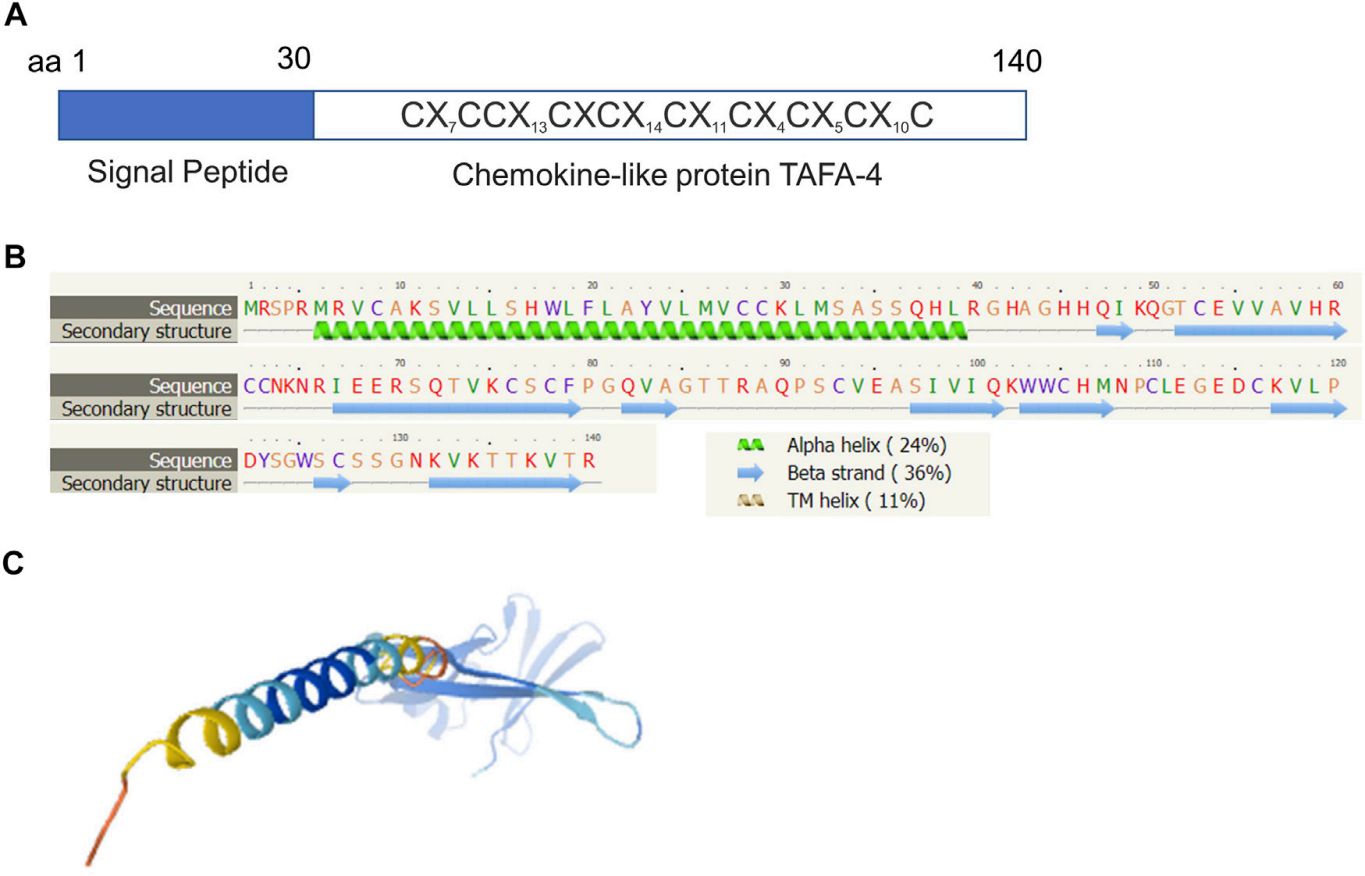
Molecular structural analysis of TAFA4. Molecular structure of TAFA4, predicted by bioinformatics information based on Uniprot (https://www.uniprot.org/), showing **(A)** a characteristic of a chemokine like ligand, **(B)** the Phyre2 web portal (http://www.sbg.bio.ic.ac.uk/phyre2/), and **(C)** AlphaFold web portal (https://alphafold.ebi.ac.uk/).

Molecular structural analyses revealed that human TAFA4 consists of a signal sequence (amino acid residues 1–30), and chemokine-like protein (amino acid residues 31–140) (Figure 4A). Secondary structural analyses predicted that TAFA4 contains an alpha helix at its N terminal region, and nine beta strands at its C terminal region by Phyre2 web portal analysis (Figure 4B) (Kelley et al., 2015). A predicted 3D structure of TAFA4 is consistent with its secondary structure features (Figure 4C) (Jumper et al., 2021).

In comparison with the structure of TAFA4, the human TAFA1 gene (also called FAM19A1) encodes a 133 amino acid (aa) precursor which consists of a signal sequence (1–19 residues) and a mature chain (20–133 residues). It has a typical motif structure of CX7CCX13CXCX14CX11CX4CX5CX10C, at the C terminal region. TAFA1 is detected in the brain, where it is most abundantly expressed in the frontal, temporal, occipital, and parietal cortices, and medulla, whilst it is expressed at low levels in the basal ganglion, thalamus, and cerebellum (Tom Tang et al., 2004).

The human TAFA2 gene (also called FAM19A2) encodes a 131aa precursor which consists of a signal sequence (1–30 residues) and a mature chain (31–131 residues). It has a typical structure of CX7CCX13CXCX14CX11CX4CX5CX10C at the C terminal region. TAFA2 is most abundantly expressed in the occipital and frontal cortices, and medulla within the CNS, followed by colon, heart, lung, spleen, kidney, and thymus (Tom Tang et al., 2004).

The human TAFA3 gene (also called FAM19A3) encodes a 133aa precursor which consists of a signal sequence (1–30 residues) and a mature chain (31–133 residues). It bears a typical structure of CX7CCX13CXCX14CX11CX4CX5CX10C at the C terminal region. TAFA3 is differentially expressed in the brain (Tom Tang et al., 2004).

The human TAFA5 gene (also called FAM19A5) encodes a 132aa precursor which consists of a signal sequence (1–43 residues) and a mature chain (44–132 residues). It has a distinct structure of CX7DSX13CXCX14CX11CX4CX5CX10C at the C terminal region. TAFA5 is most abundantly expressed in the basal ganglia and cerebellum (Tom Tang et al., 2004).

Overall, FAM19A genes are expressed during CNS development and in the postnatal brain (Tom Tang et al., 2004; Sarver et al., 2021). Utilizing the Genevisible®- based bioinformatics tool (Hruz et al., 2008), comparative analyses of TAFA1-4 gene expressions showed that they are predominantly in the spinal cord, retina, lens and iris (data not shown).

## Molecular Structure and Expression of TAFA4 Receptor FPR1

**FIGURE 5:**
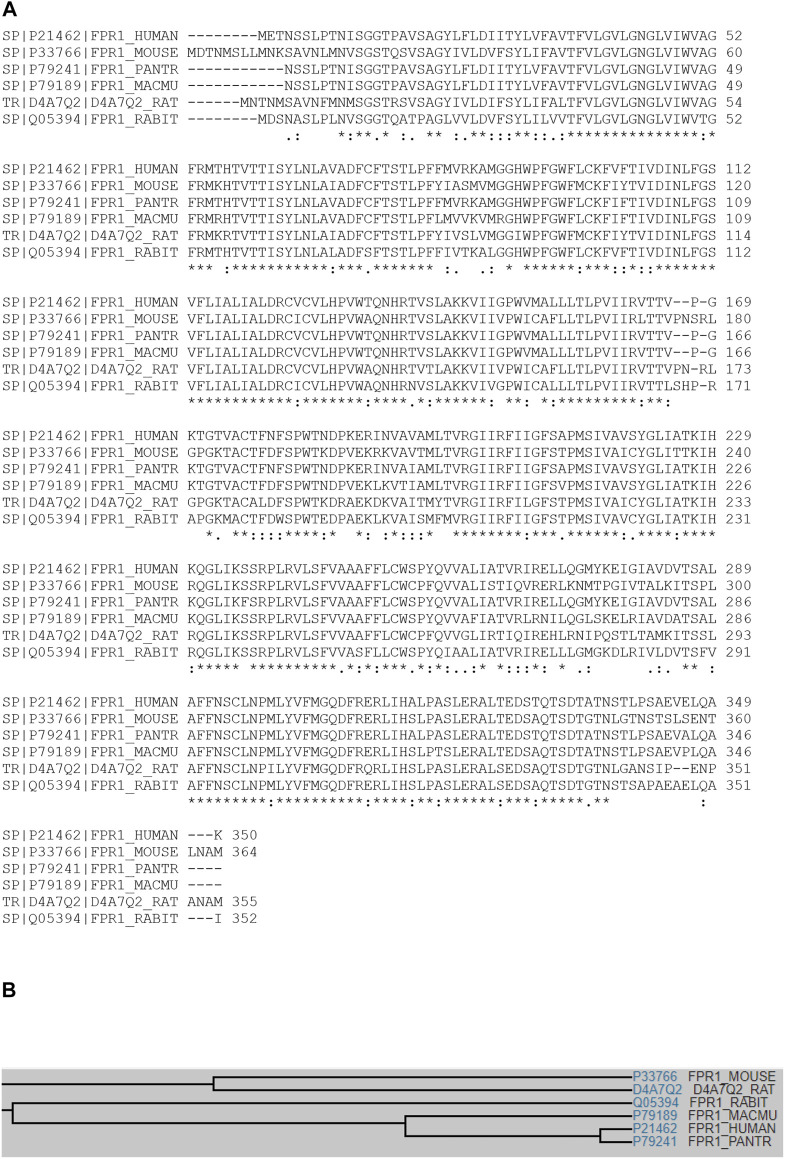
Multiple sequence alignment of FPR1. **(A)** Multiple sequence alignment analyses show that FPR1 shares sequence identity and similarity with FPR1 homologues among various species including human, mouse, chimpanzee, Rhesus macaque, rat, and rabbit (https://www.uniprot.org/align). **(B)** A family tree of FRP1 homologue proteins is elucidated.

**FIGURE 6:**
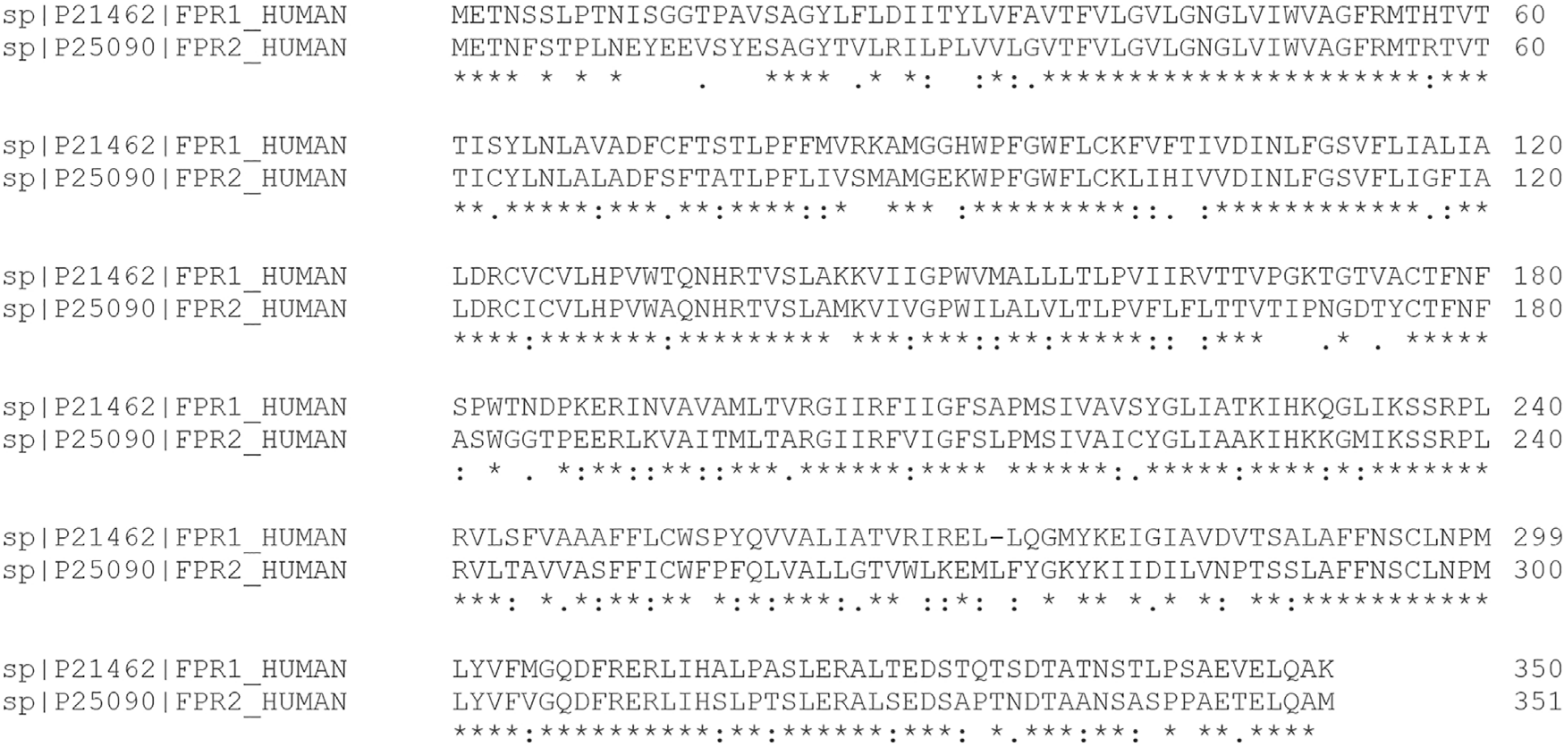
Comparative sequence alignment of FPR1and FPR2. Multiple sequence analysis demonstrates that human FRP1 and FPR2 share significant degree of amino acid sequence identity.

FPR1 belongs to the G-protein-coupled seven-transmembrane receptor superfamily (Yamagami et al., 1994). It exhibits sequence identity among species, including human, mouse, chimpanzee, rhesus macaque, rat, and rabbit (Figure 5A) with a common family tree (Figure 5B). FPR1 also shares sequence homology and conserved cysteine residues with FPR2 (Figure 6).

**FIGURE 7:**
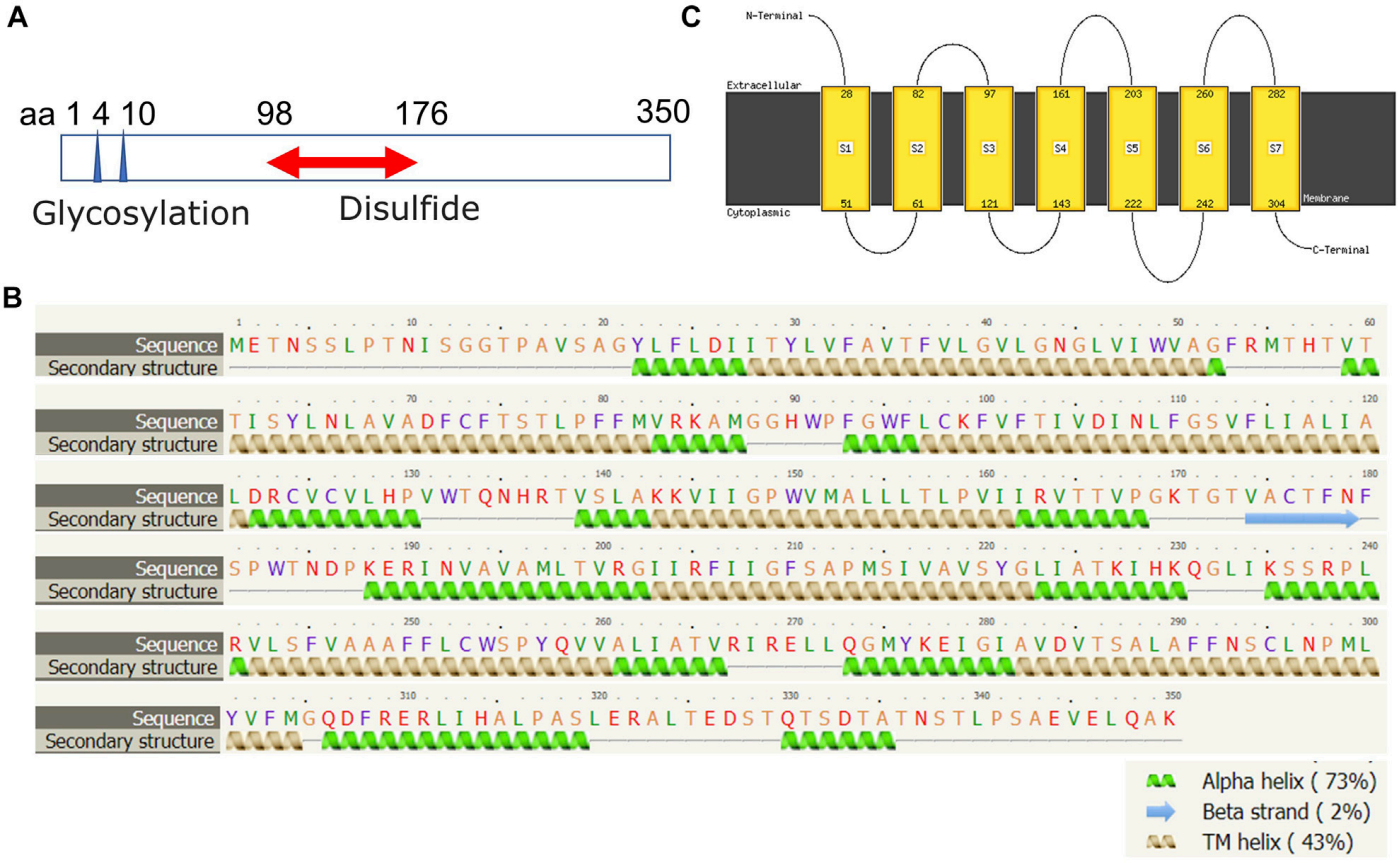
Molecular structural analysis of FPR1. Molecular structure of FPR1, predicted by bioinformatics information based on **(A)** Uniprot (https://www.uniprot.org/), **(B,C)** the Phyre2 web portal (http://www.sbg.bio.ic.ac.uk/phyre2/) all showing characteristics of G protein-coupled receptor.

**FIGURE 8:**
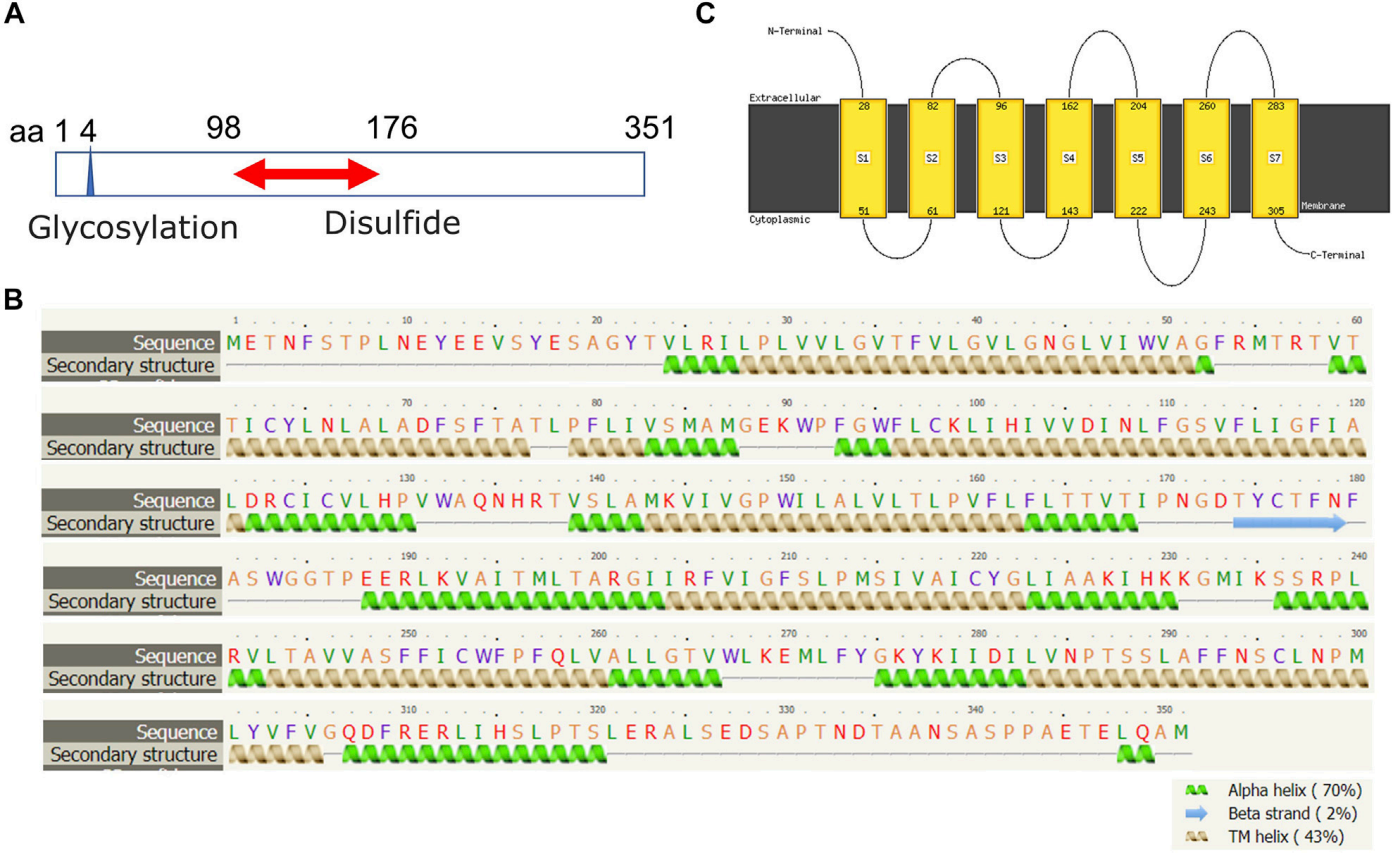
Molecular structural analysis of FPR2. Molecular structure of FPR2, predicted by bioinformatics information based on **(A)** Uniprot (https://www.uniprot.org/), **(B,C)** the Phyre2 web portal (http://www.sbg.bio.ic.ac.uk/phyre2/) all showing characteristics of G protein-coupled receptor.

**FIGURE 9:**
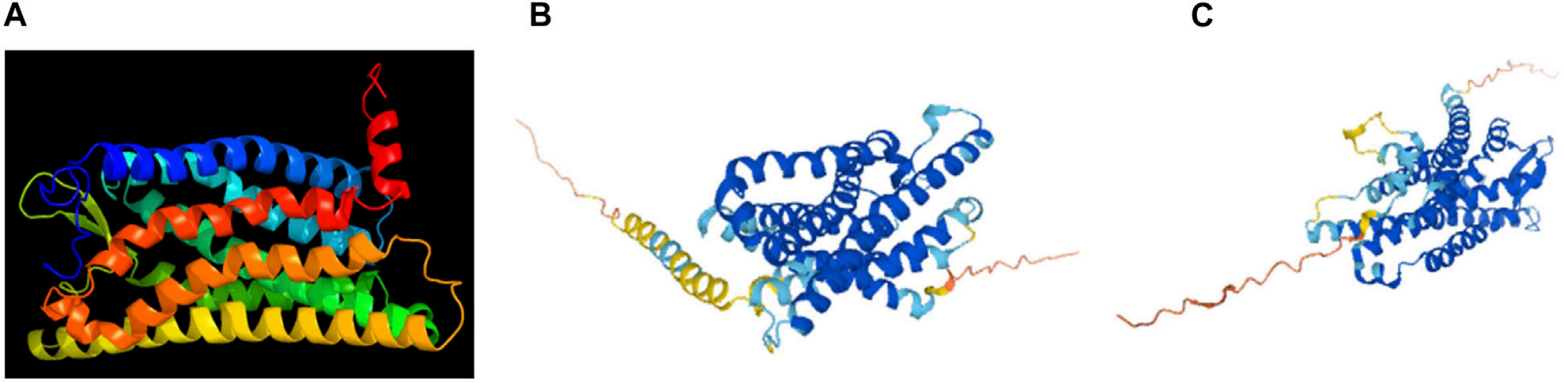
3D structural images of FRP1 and FPR2. 3D structure of FRP1 and FPR2, predicted by **(A)** the Phyre2 web portal (http://www.sbg.bio.ic.ac.uk/phyre2/), **(B)** 3D structure of FRP1 and **(C)** FPR2 predicted by AlphaFold web portal (https://alphafold.ebi.ac.uk/).

The human FPR1 gene encodes a 350aa protein that is predicted to consist of two putative N-linked glycosylation residues at positions 4 and 10, and a disulphide bond at residues 98 ↔ 176 (Figure 7A). It also consists of three putative phosphoserine sites (residues 328, 332, and 338) and four putative phosphothreonine sites (residues 329, 331, 334, 336, and 339). It has multiple alpha helices and beta strands (Figure 7B) and seven transmembrane domains (Figure 7C) as predicted by the Phyre2 web portal (Kelley et al., 2015). Similarly, the human FPR2 gene encodes a 351aa protein that is predicted to consist of one putative N-linked glycosylation at position 4, and a disulphide bond at residues 98 ↔ 176 (Figure 8A). It too has multiple alpha helices and beta strands (Figure 8B) and seven transmembrane domains (Figure 8C) as predicted by the Phyre2 web portal (Kelley et al., 2015). Additional Phyre2 web portal analyses show that the 3D structures of both FPR1 and FPR2 were predicted to have one common structure (Figure 9A) (Kelley et al., 2015). The 3D structures of FPR1 (Figure 9B) and FPR2 (Figure 9C) were also predicted by AlphaFold analyses to be consistent with their secondary features (Jumper et al., 2021).

**FIGURE 10:**
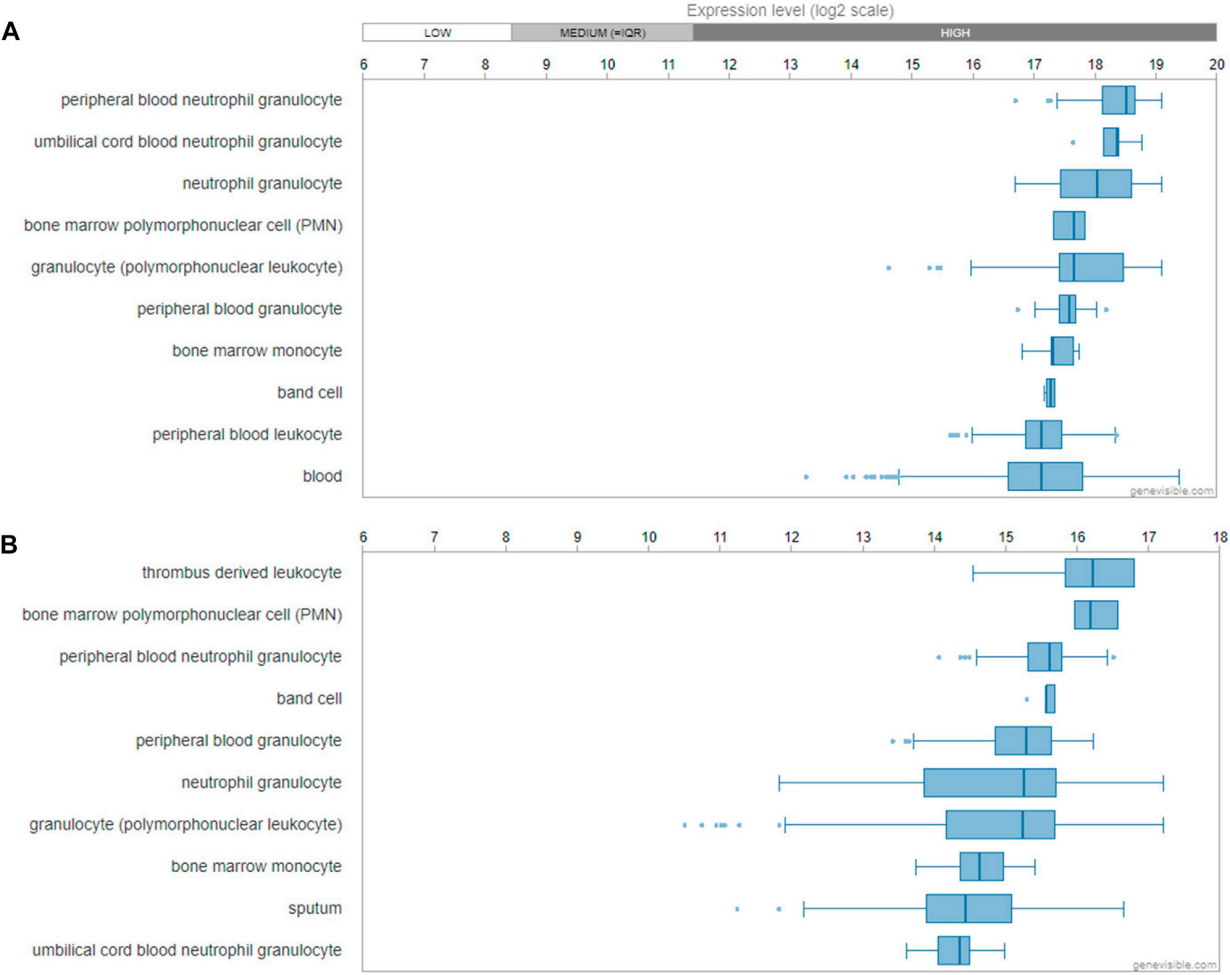
Gene expression profiling of FPR1 and FPR2. mRNA expression profiling of FPR1 **(A)** and FPR2 **(B)** gene in human tissues and cells predicted by Genevisible^®^ bioinformatics tool (http://genevisible.com), with the ten most highly ranking mRNA express levels, respectively.

Utilizing the Genevisible®- based bioinformatics tool (Hruz et al., 2008), human FPR1 mRNA was found most abundantly expressed in peripheral blood neutrophil granulocyte, umbilical cord blood neutrophil granulocyte, neutrophil granulocyte and bone marrow polymorphonuclear cell (Figure 10A). Similarly, human FPR2 mRNA was revealed to be most highly expressed in thrombus derived leukocyte, bone marrow polymorphonuclear cell, peripheral blood neutrophil granulocyte, band cell, and peripheral blood granulocyte (Figure 10B).

## The Roles of TAFA4 and FPR1 in the Spinal Cord

The spinal cord is a vital tubelike structure originating from the brain stem and extending the length of the spinal column for the transmission of motor and sensory nerve signals, which are vitally important for the control of functions, such as reflexes and coordinated physical movements like walking. Spinal cord injury results in neurologic pain and disability. Nociceptors detect intense, noxious, painful stimuli and are present in a subpopulation of peripheral nerves with cell bodies located in DRG (body) and the trigeminal ganglion (face) (Basbaum et al., 2009). Nociception occurs when nociceptors become excited by a stimulus (chemical, thermal, or mechanical) that reaches a noxious intensity and the sensation of pain is evoked (Basbaum et al., 2009). TAFA4 (or FAM19A4) was shown to be a marker of C-low-threshold mechanoreceptors (C-LTMRs), which are unique among sensors of touch, appearing to transmit both sensations of pleasantness and injury-induced mechanical pain (Seal et al., 2009; Delfini et al., 2013). Findings also suggest that TAFA4+ neurons exhibit intrinsic features of mechano-nociceptors (Delfini et al., 2013). Further, TAFA4-null mice showed elevated hypersensitivity to mechanical and chemical stimuli, and excitability of spinal cord lamina IIi neurons in response to inflammation and nerve injury, whereas administration of recombinant TAFA4 in C57/Bl6 mice *via* an injection into the spinal canal was able to reverse carrageenan-induced mechanical hypersensitivity (Delfini et al., 2013). C-LTMR-derived TAFA4 is therefore postulated to modulate neuron excitation and to regulate the somatic sensation threshold which indicates it could be a potent analgesic agent (Delfini et al., 2013). TAFA4 is consistently reported to have an analgesic role in pain relief (Delfini et al., 2013; Reynders and Moqrich, 2015) and has been implicated in regulating spinal cord pain (Delfini et al., 2013; Gaillard et al., 2014; Kambrun et al., 2018). Using a knock-in mouse, TAFA4 was predominantly localised in C-LTMRs neurons in DRGs (Salio et al., 2021). Recent research further investigated the function of TAFA4 in modulating neuronal excitation and synaptic transmission (Kambrun et al., 2018). C-LTMR-derived TAFA4 was shown to enhance the inhibition of synaptic transmission from high-threshold C-fibres of spinal networks by depressing local excitatory synapses (Kambrun et al., 2018). Using an animal model of inflammation, findings from this study revealed that mechanical pain and neuronal response to a noxious stimulus was alleviated by TAFA4, which appears to be mediated by γ-aminobutyric acid (GABA)-ergic transmission (Kambrun et al., 2018). These results demonstrated the integral function of TAFA4 in response to inflammation by the promotion of microglial retraction and the increased number of inhibitory synapses on lamina IIi somata (Kambrun et al., 2018). Conclusively, GABAergic interneurons appear to be a primary signal integration relay for C-LTMRs, and the release of TAFA4 by C-LTMRs is suggested to inhibit nociceptive excitatory synaptic transmission in the spinal cord resulting in the alleviation of pain in pathogenic conditions (Kambrun et al., 2018). However, direct evidence to indicate FPR1/2 expression can be upregulated in DRG or spinal cord is lacking.

Most recently, TAFA4 was shown to relieve injury-induced mechanical hypersensitivity by restoring spinal neuron activity to normal, which is further suggestive of its therapeutic potential (Yoo et al., 2021). TAFA 4 was found to reverse inflammatory, postoperative, and spared nerve injury (SNI)-induced mechanical pain in mice (Yoo et al., 2021). Mechanistically TAFA4 was able to reverse injury-induced A-type K^+^ (I_A_) current decreases in spinal lamina II outer excitatory interneurons (L-IIo ExINs) and was also able to simultaneously reverse SNI-induced increases in I_A_ and decreases in hyperpolarization-activated current (I_h_) in lamina II inner inhibitory interneurons (L-IIi InhINs) (Yoo et al., 2021). The rescue of ion current changes to normal levels in both IN groups was dependent on low-density lipoprotein receptor-related proteins (LRPs) (Yoo et al., 2021). TAFA4 therefore is effective at reversing injury-induced mechanical hypersensitivity and is a prime therapeutic target for the treatment of injury-induced mechanical pain. Leading recent research identified a neuroimmune regulatory pathway which is promoted by TAFA4 and has potential novel therapeutic applications for inflammatory diseases (Hoeffel et al., 2021). TAFA4 derived from sensory neurons was shown to promote macrophage coordinated tissue repair (Hoeffel et al., 2021). TAFA4 was found to be expressed by C-LTMRs, a subset of Gα_i_-Interacting Protein (GINIP) positive neurons in the skin, and to mediate the inflammatory profile of tissue-resident macrophages in a mouse model of sunburn skin damage (Hoeffel et al., 2021). Conditional ablation of sensory neurons expressing GINIP resulted in defective tissue regeneration and in dermal fibrosis (Hoeffel et al., 2021). Whilst deletion of Tafa4 in mice resulted in diminished expression of IL-10 by T-cell immunoglobulin and mucin domain containing 4 (TIM4) positive dermal macrophages after UV-induced skin damage, which was accompanied by increased skin inflammation, impaired healing, poor tissue regeneration, and dermal fibrosis (Hoeffel et al., 2021). Mechanistically these results suggest that TAFA4 is produced by C-LTMRs following UV-induced skin damage, which increases the production of IL-10 by tissue-resident macrophages and the survival of tissue-repair macrophages, leading to diminished skin inflammation, the prevention of tissue fibrosis, and enhanced tissue regeneration (Hoeffel et al., 2021). Additionally, reduced GABAergic inhibition is characteristic of neuropathic pain and previous research identified the involvement of GINIP in peripheral GABA type B (GABAB) receptor nociceptive signal transmission (Gaillard et al., 2014). GINIP null mice showed impaired responsiveness to GABAB, an inhibitory neurotransmitter, and developed prolonged and specific hypersensitivity when tested by inflammation and neuropathy models (Gaillard et al., 2014). Together with subsequent findings suggesting a protective role of TAFA4 for injury-induced by mechanical and chemically induced neuro-inflammatory pain (Kambrun et al., 2018), TAFA4 consistently emerges as a promising therapeutic target for pain relief, and tissue repair and regeneration. Future research is necessary to develop the therapeutic potential of TAFA4 for the alleviation of pain from neuro-inflammatory disorders and injury-induced mechanical pain.

**FIGURE 11:**
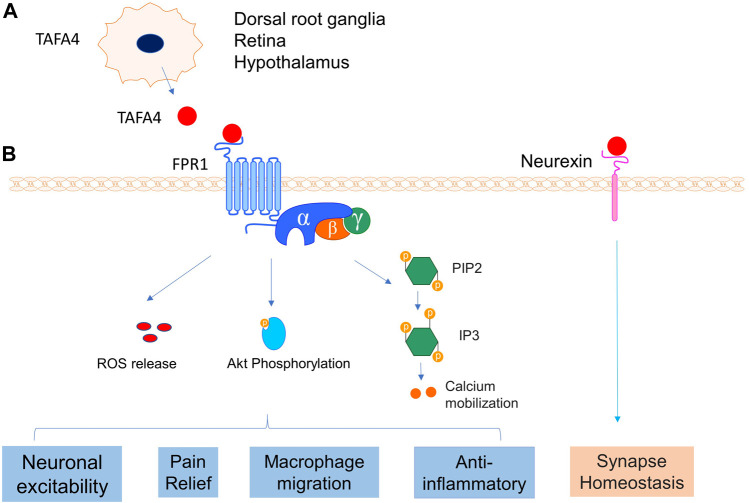
TAFA4 and its receptors interaction. **(A)** TAFA4is expressed in DRG, retina and hypothalamus. **(B)** TAFA4 binds FPR1 and activates signalling pathways of Akt phosphorylation, calcium mobilization, and ROS release, leading to various cellular activities. In addition, TAFA4 binds cell surface neurexin and mediates synapse homeostasis.

Previous research has identified TAFA4 as a cytokine ligand of FPR1 for the inflammatory response to pathogenic infections (Wang et al., 2015). TAFA4 is upregulated in monocytes and macrophages when induced by LPS, and FPR1 was identified as a receptor for TAFA4 *via* ligand binding and blockage, and receptor internalization assays (Wang et al., 2015). This study also showed that TAFA4 was able to induce chemotactic activities on macrophages and enhance macrophage phagocytosis, which occurred with increased phosphorylation of protein kinase B (Akt) (Wang et al., 2015). TAFA4 therefore appears to play a critical role in the chemoattraction and phagocytosis of macrophages, and ROS release following an infection (Figure 11) (Wang et al., 2015). Several studies of TAFA family interactions have provided insights of the molecular signalling mechanisms of TAFA4. For example, findings from these studies reveal that binding of TAFAs to FPR1 and FPR2 led to the activation of the phosphatidylinositol-calcium second messenger system and intracellular calcium mobilization (Murphy and McDermott, 1991; Postma et al., 2004; Prat et al., 2009). FAM19A1-A4 (or TAFA1-4) could also interact with the cysteine-loop domain of neurexins and presynaptic adhesion molecules *via* intermolecular disulphide bonds (Khalaj et al., 2020). The molecular events of signalling activation following ligand receptor bindings of TAFA4 remain incompletely known. FAM19As are thought to interact with cell surface neurexins to mediate signalling across the synapse through connecting presynaptic and postsynaptic neurons (Figure 11) (Khalaj et al., 2020; Sarver et al., 2021). Together further research is needed to illuminate whether FAM19A interactions are cell type specific both for FPR1 and FPR2 and define the cellular signaling pathways and molecular mechanisms of TAFA4, which is vitally important for the development of therapeutic applications targeting TAFA4.

## The Emerging Role of Other TAFAs in the Neural System and Disorders

FAM19As are predominantly expressed in specific subtypes of neurons and serve as cell type-specific regulators of neurexin modifications (Tom Tang et al., 2004; Khalaj et al., 2020). The nomenclature of human TAFAs and FAM19As are summarized in Table 1. Among five subfamily members of FAM19As/TAFAs proteins, the role of TAFA4 is relatively well characterised, whilst the role of other TAFAs is emerging.

**TABLE 1:** Nomenclature of human TAFAs and FAM19A

Name	Full name	Other name	Uniprot no.
TAFA1	Chemokine-like protein TAFA-1	FAM19A1	Q7Z5A9
TAFA2	Chemokine-like protein TAFA-2	FAM19A2	Q8N3H0
TAFA3	Chemokine-like protein TAFA-3	FAM19A3	Q96LR4
TAFA4	Chemokine-like protein TAFA-4	FAM19A4	Q96LR4
TAFA5	Chemokine-like protein TAFA-5	FAM19A5	Q7Z5A7

For instance, FAM19A1 has recently been linked with regulating food intake and behaviour. Mice with AM19A1 deficiency revealed that male FAM19A1 KO mice displayed more altered food intake patterns during the light and dark cycle, accompanied with hyperactive, and locomotor hyperactivity than female KO mice (Lei et al., 2019). On the other hand, female FAM19A1 KO mice showed reduced anxiety and sensitivity to pain, associated with elevated norepinephrine and dopamine turnover in the striatum, suggesting sex-dependent phenotypes between male and female (Lei et al., 2019). Consistently, mice with FAM19A1 deficiency exhibited hyperactive locomotor behaviour, long-term memory deficits and fear acquisition failure (Yong et al., 2020). Using a LacZ reporter gene system, FAM19A1 was shown to be expressed in the pyramidal cells of cortical layers during brain development in mouse (Yong et al., 2020). In a study using hippocampal cultures, FAM19A1 was found to reduce neurexin O-glycosylation and heparan sulphate modification, suggesting that FAM19A1 is involved in the post-translational modification and function of neurexins (Khalaj et al., 2020). Collectively, these findings suggest that FAM19A1 plays a role in neurodevelopment and mature brain function (Yong et al., 2020).

Whilst TAFA2 is suspected to be a gene involved with the development of intellectual deficiency and mental retardation. Wang X. et al. (2018) reported that TAFA2 gene knockout mice exhibited impaired spatial learning and memory and increased level of anxiety-like behaviours. Further, TAFA2 deficiency resulted in severe neuronal reduction and increased apoptosis in the brain with downregulation of PI3K/Akt and MAPK/Erk pathways with down regulation of Brain-derived neurotrophic factor (BDNF), c-fos and Neurofibromin 1(NF1), and CREB-binding protein (CBP) gene expression, suggesting that TAFA2 acts as a neurotrophic factor essential for neuronal survival and neurobiological functions (Wang X. et al., 2018). More recently, FAM19A2/TAFA-2 was found to regulate metabolic activities and food intake. Administration of FAM19A2/TAFA-2 to mice just before the initiation of dark period led to increased food intake and meal number, but reduced meal size (Okada et al., 2019). Outside the nervous system, TAFA2 was found to increase the migration and motility of mesenchymal stem cells (MSC), and to be involved with the recruitment of MSC to bone fracture sites. It was revealed that TAFA2 was able to promote MSC migration through activation of the Rac1-p38 pathway (Jafari et al., 2019). In addition, serum levels of TAFA2 in patients was also increased after hip fracture (Jafari et al., 2019). TAFA2 gene expression was found to be upregulated by interleukin-1β and during the inflammatory phase of fracture healing in mice (Jafari et al., 2019). The pleiotropy of TAFA2 therefore requires further investigation to uncover its diverse roles by a tissue-specific approach. Little is known about the function of TAFA3. For example, TAFA-3 appears to be a signalling molecule of the pars tuberalis, which is postulated to be a control centre of changing seasonal function rhythms (Fischer et al., 2012; Korf, 2018). Additionally, FAM19A3 was found to be upregulated in microglia of a middle cerebral artery occlusion (MCAO) mouse model *via* modulating the microglia/macrophage polarization dynamics and to ameliorate cerebral ischemia (Shao et al., 2015). Further research is needed to advance our knowledge and understanding of TAFA3 function.

TAFA5 has recently been implicated in the progression of cognitive impairment in vascular dementia patients and could be a vital regulator of depression (Huang et al., 2020; Li et al., 2020). TAFA5 was found to be predominantly expressed in the hypothalamic paraventricular nucleus (PVN) (Paulsen et al., 2008). Consistently, FAM19A5 was found to be highly expressed in the embryonic hippocampus (Huang et al., 2020). More recently, it was revealed that the hypothalamic expression of TAFA5/FAM19A5 was induced by TNF-α, thereby suggesting its involvement with inflammatory responses of the hypothalamus (Kang et al., 2020). Interestingly, FAM19A5 knockdown led to the reduction of TNF-α-induced anorexia and decreased loss of body weight, whereas administration of FAM19A5 by an intracerebroventricular route resulted in anorexia, body weight loss, hyperthermia, and the enhanced expression of inflammatory factors (Kang et al., 2020). Further, FAM19A5 administration also resulted in increases in the activation of c-fos and the expression of preproopiomelanocortin (POMC) in POMC neurons in hypothalamus, suggesting that FAM19A5 plays a part in inflammatory responses of the hypothalamus (Kang et al., 2020). Mice with FAM19A5 gene deletion demonstrated depressive-like behaviours and impaired hippocampus-dependent spatial memory, which was accompanied by decreased expression of alpha-amino-3-hydroxy-5-methyl-4-isoxazolepropionic acid receptors and N-methyl-D-aspartic acid receptors (Huang et al., 2020). Mice additionally showed reduced glutamate release and neuronal activity in the hippocampus, and the decreased density of dendritic spines (Huang et al., 2020). These data suggest that FAM19A5 regulates depression and spatial cognition *via* the hippocampus (Huang et al., 2020). In line with this finding, the serum FAM19A5 level of vascular dementia patients was found to be increased, suggestive of a role in cognitive impairment of vascular dementia patients (Li et al., 2020). Together, these findings indicate TAFA5 could be a critical regulator of cognitive function and warrants further research of its potential for diagnosis and treatment-based approaches of depression.

Beyond the nervous system, research indicates that downregulation of FAM19A5 during obesity could lead to the development of cardiometabolic diseases (Wang Y. et al., 2018). In this study, FAM19A5 was found to inhibit postinjury neointima formation *via* sphingosine-1-phosphate receptor 2-G12/13-RhoA signalling (Wang Y. et al., 2018). In addition, serum FAM19A5 is postulated to be a biomarker of cardio-metabolic disease, as it was found to be positively correlated with risk factors, such as waist circumference, waist-to-hip ratio, alanine aminotransferase, fasting plasma glucose, glycated haemoglobin and mean brachial-ankle pulse wave velocity (Lee et al., 2019). Whereas in the skeletal system, FAM19A5 was found to inhibit osteoclastogenesis *via* FPR2, suggesting a role of TAFA family proteins in bone disorders (Park et al., 2017). The pleiotropy of TAFA5 therefore requires further investigation to elucidate the diverse roles of TAFA5 in a disease-specific based approach.

TAFA5 has also been implicated in cancers (Diaz de Stahl et al., 2005; Hu et al., 2019). TAFA5 was upregulated in gastric cancer cells compared with normal cells, which is correlated with unfavourable patient prognoses. Overexpression of TAFA5 in gastric cancer was associated with poor differentiation, tumour progression, nodal, and metastasis stages. *In vitro* findings showed that downregulation of TAFA5 could inhibit the proliferation and migration of gastric cancer cell lines. Interestingly, TAFA5 expression was significantly correlated with genes found to be associated with epithelialmesenchymal transition, which is suggestive of TAFA5 involvement in the progression of gastric cancer (Hu et al., 2019). In addition, previous studies have found that TAFA5 was associated with DNA copy number alterations in gliomas but its role of in tumour development requires further investigation (Diaz de Stahl et al., 2005).

## Summary

The TAFA family consists of five members TAFA1–5 (also known as FAM19A1–5). They act as secretory chemokine like ligands or neurokines and exhibit pleiotropic functions in various tissues. TAFA4 was first reported to have an analgesic role in pain relief (Delfini et al., 2013; Reynders and Moqrich, 2015). The TAFA4 gene is predominantly expressed in the CNS. As a secreted ligand, TAFA4 binds to cell surface receptor FPR1 or neurexin to mediate versatile physiological processes including food intake, learning and memory, behaviours, locomotor activity, pain relief, and synapse homeostasis. Substantial recent research has shown the effectiveness of TAFA4 for the alleviation of pain caused by mechanical injury, neuro-inflammatory disorders, and chemical or infectious stimuli. Further research is required to completely characterise the molecular mechanisms and cellular signalling pathways of TAFA4 for the development of therapeutic applications targeting a range of pathogenic conditions. Advancing knowledge of the selective expression, regulation, and diverse biological functions of TAFA4 and FAM19A members in a tissue tropism dependent manner will enable us to develop strategies to treat TAFA4 and FAM19A related disorders, such as spinal cord injury and neuropathic pain.
